# Diagnostic performance of dynamic electrocardiography in the diagnosis of myocardial ischemic attack in coronary heart disease: a systematic review and meta-analysis

**DOI:** 10.3389/fmed.2025.1646417

**Published:** 2025-09-17

**Authors:** Wenming Lv

**Affiliations:** Department of Special Inspection, The Affiliated People’s Hospital of Ningbo University, Ningbo, China

**Keywords:** coronary heart disease, myocardial ischemia, dynamic electrocardiography, diagnostic performance, meta-analysis

## Abstract

**Background:**

Coronary heart disease (CHD) remains a leading cause of mortality worldwide, highlighting the need for early and accurate diagnosis of myocardial ischemia to improve patient outcomes. Dynamic electrocardiography (ECG) has become a critical diagnostic tool due to its capacity for continuous cardiac electrical activity monitoring. However, existing studies show considerable variation in its diagnostic performance. To establish higher-level evidence, this study systematically evaluates the diagnostic accuracy of dynamic ECG for myocardial ischemic episodes in patients with CHD through a comprehensive systematic review and meta-analysis.

**Methods:**

A comprehensive literature search was conducted through May 2025 using PubMed, Embase, Web of Science, Cochrane Library, China National Knowledge Infrastructure, and Wanfang databases. Study quality was assessed using the QUADAS-2 instrument. Diagnostic performance was evaluated using sensitivity, specificity, positive likelihood ratio (PLR), negative likelihood ratio (NLR), diagnostic odds ratio (DOR), and the area under the receiver operating characteristic curve (AUC).

**Results:**

The meta-analysis included 24 studies comprising 3,509 participants. The pooled diagnostic performance of dynamic ECG for myocardial ischemia showed a sensitivity of 0.75 (95% CI: 0.70–0.80), specificity of 0.70 (95% CI: 0.64–0.75), PLR of 2.50 (95% CI: 1.99–3.13), NLR of 0.36 (95% CI: 0.28–0.45), DOR of 6.64 (95% CI: 4.55–9.69), and an AUC of 0.79 (95% CI: 0.75–0.82). Subgroup analyses indicated higher diagnostic accuracy in studies involving confirmed CHD cases and those using coronary angiography as the reference standard.

**Conclusion:**

Dynamic ECG exhibits moderate diagnostic value for detecting myocardial ischemia in patients with CHD. Its clinical application should be integrated with complementary diagnostic approaches. Further high-quality research is necessary to confirm its diagnostic utility and refine implementation protocols.

**Systematic review registration:**

INPLASY202560026.

## Introduction

Coronary heart disease (CHD) is a pathological condition characterized by coronary artery stenosis or occlusion caused by atherosclerosis. Acute cardiovascular events triggered by CHD remain the leading cause of mortality worldwide (1). According to the 2023 Report on Cardiovascular Health and Diseases in China, the number of patients with CHD has reached 11.39 million. The disease burden continues to grow, driven by an aging population and the increasing prevalence of metabolic disorders (2). Myocardial ischemia—the central clinical manifestation of CHD—results from an imbalance between coronary blood supply and myocardial oxygen demand, leading to insufficient oxygen delivery to cardiac tissue (3). Without timely intervention, transient ischemia may quickly evolve into acute coronary syndrome, significantly elevating the risk of sudden cardiac death (4). Accordingly, the development of reliable early diagnostic tools for myocardial ischemia is critical to improving outcomes and optimizing healthcare resource allocation.

Among current diagnostic modalities, dynamic electrocardiography (ECG) is widely employed for out-of-hospital myocardial ischemia monitoring due to its non-invasive nature and accessibility (5). Compared to conventional resting ECG, which captures only brief electrical changes, dynamic ECG enhances the detection of transient ST-T segment abnormalities through continuous 24–72-h recording, making it particularly effective for outpatient populations experiencing paroxysmal chest pain (6). Additionally, its ability to monitor cardiac activity in real time during daily life facilitates the identification of associations between myocardial workload and ischemic episodes, supporting personalized treatment approaches (7).

However, the diagnostic performance of dynamic ECG for myocardial ischemia remains controversial. Several studies have demonstrated substantial variability in sensitivity and specificity, largely attributable to: (1) bias in baseline characteristics of study populations (e.g., age distribution, comorbid conditions such as diabetes); (2) lack of standardized diagnostic thresholds (e.g., differing criteria for ST-segment depression ≥0.1 mV or ≥1 mm, lasting ≥1 min); (3) technical inconsistencies (lead configuration, motion artifact recognition); and (4) divergent reference standards (coronary angiography, myocardial perfusion imaging, or intravascular ultrasound) (8). These methodological inconsistencies hinder clinical interpretation and limit the development of evidence-based guidelines.

To address these limitations and provide a comprehensive assessment of diagnostic performance, this systematic review and meta-analysis aims to synthesize available evidence on dynamic ECG for detecting myocardial ischemic episodes in patients with CHD. By pooling results across studies, we evaluate its diagnostic accuracy, explore sources of heterogeneity, and identify key factors influencing performance.

## Methods

### Data sources, search strategy, and selection criteria

This systematic review and meta-analysis was conducted in accordance with the updated 2020 Preferred Reporting Items for Systematic Reviews and Meta-Analyses (PRISMA) guidelines (9). Our study was registered in INPLASY platform (number: INPLASY202560026). We included studies that evaluated the diagnostic performance of dynamic ECG monitoring for myocardial ischemia in patients with CHD, regardless of language. A globally comprehensive search was conducted through May 2025 across both international (PubMed, Embase, Web of Science, Cochrane Library) and Chinese (China National Knowledge Infrastructure, Wanfang) databases to minimize geographic bias. The search strategy combined terms covering dynamic ECG, coronary heart disease, myocardial ischemia, and diagnosis (detailed in Supplementary File 1). We explicitly included non-Chinese language studies and applied no regional restrictions. Additionally, we manually reviewed reference lists of relevant articles and reviews to identify additional eligible studies not captured in the database search.

Two reviewers independently conducted title/abstract screening and full-text evaluation using a standardized form. Discrepancies were first resolved through structured discussion, with explicit documentation of reasoning. For studies consensus was not reached after two rounds of discussion, a third senior reviewer, arbitrated by re-evaluating the original study against inclusion criteria and providing a final decision, which was documented in a conflict resolution log. Inclusion criteria were: (1) population: patients with confirmed or suspected CHD; (2) diagnostic tool: 12-lead dynamic ECG monitoring; (3) outcome: reported diagnostic data for myocardial ischemia with a complete contingency table (true positives, false positives, true negatives, false negatives); and (4) no restriction on study design. Studies were excluded if they: (1) included <10 participants; (2) lacked extractable diagnostic accuracy data; (3) involved non-human participants; (4) provided insufficient methodological detail for quality assessment; or (5) employed non-standard ECG configurations (<12 leads or modified placements).

### Data collection and quality assessment

A dual verification process was used: (1) independent re-extraction after a 24-h interval; (2) cross-checking numerical data with original sources; and (3) resolving discrepancies through documented consensus meetings. For data extraction discrepancies, unresolved disagreements after discussion were escalated to the third senior reviewer, who verified the original study data and made a final determination, with all decisions recorded in a data extraction audit trail. Extracted information included: first author’s surname, publication year, geographical region, sample size, mean age, male proportion, clinical status, reference standard, diagnostic performance metrics (true positives, false positives, true negatives, false negatives). For QUADAS-2 quality assessment, two reviewers independently rated each domain (patient selection, index test, reference standard, flow/timing) as “low,” “high,” or “unclear” risk of bias (10). Disagreements in 15% of domain ratings were first addressed through discussion. Unresolved cases were reviewed by the third senior reviewer, who re-evaluated the study methodology against QUADAS-2 criteria, provided a final rating, and documented the rationale in a quality assessment log.

### Statistical analysis

This study employed true positive, false positive, true negative, and false negative data to derive key diagnostic metrics including sensitivity (detection capability of true positives), specificity (capacity to correctly exclude non-cases), positive likelihood ratio (PLR), negative likelihood ratio (NLR), diagnostic odds ratio (DOR), and the area under the receiver operating characteristic curve (AUC). Pooled estimates were generated using bivariate generalized linear mixed models with random effects to account for between-study variability (11, 12). Heterogeneity was assessed using the *I^2^* statistic and Cochran’s Q-test, with *I^2^* ≥ 50.0% or Q-test *p* < 0.10 indicating significant heterogeneity (13, 14). Prespecified subgroup analyses were performed based on disease status (confirmed vs. suspected CHD) and reference standard methodology. Publication bias was evaluated through funnel plots and Deeks’ asymmetry test (15). All meta-analytic outcomes were interpreted through two-tailed statistical testing with an *α*-level threshold of 0.05. The complete analytical workflow was executed using STATA version 14.0 (StataCorp LP, College Station, TX, United States), ensuring methodological reproducibility through standardized scripting protocols.

## Results

### Literature search

**Figure 1:**
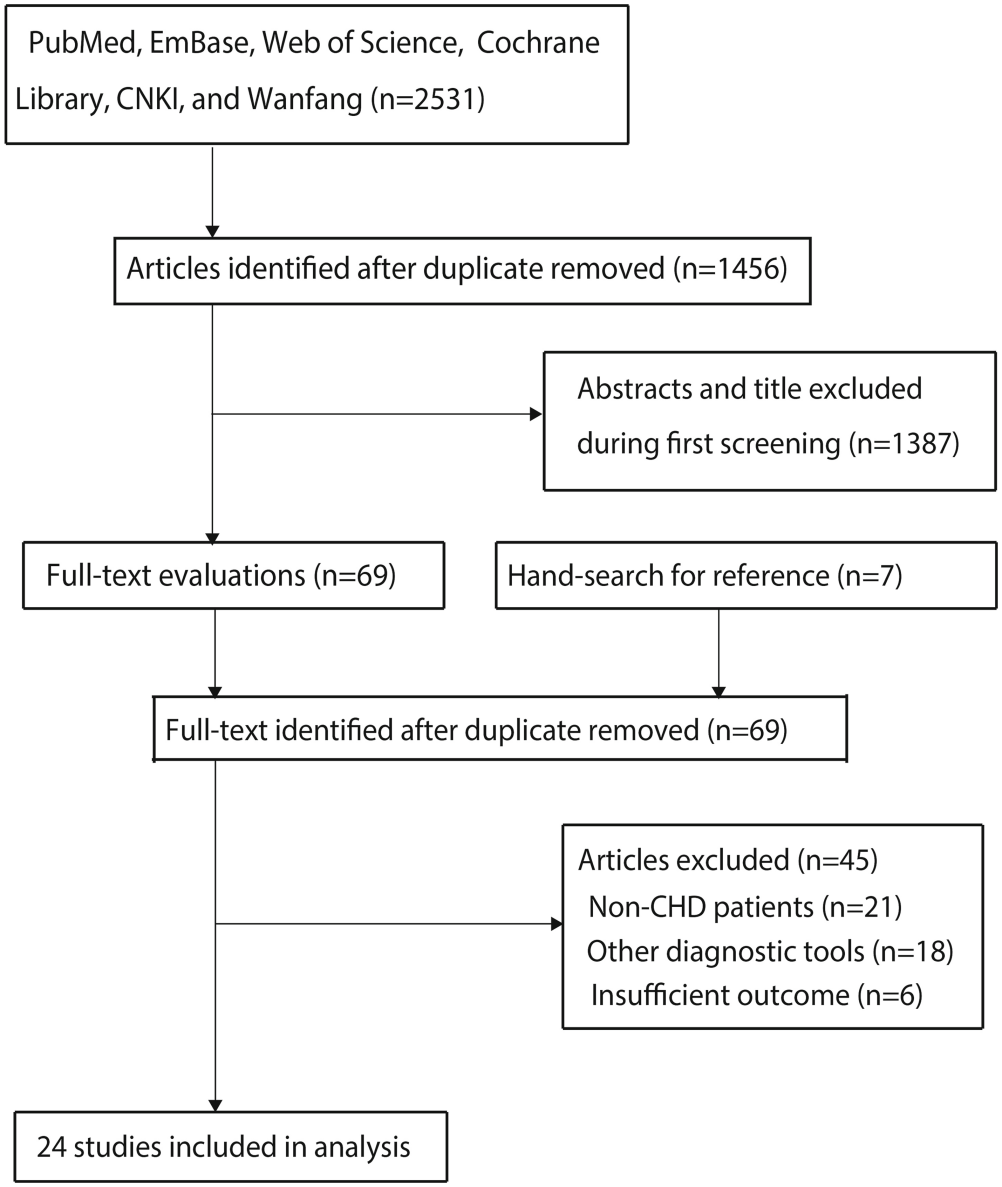
Flow diagram of the literature search and study selection process.

The electronic database search initially identified 2,531 studies, with 1,456 retained after duplicate removal. Title and abstract screening excluded 1,387 studies, leaving 69 for full-text evaluation. Following detailed assessment, 45 studies were excluded due to: (1) non-CHD populations (*n* = 21); (2) alternative diagnostic tools (*n* = 18); and (3) insufficient outcome reporting (*n* = 6). Ultimately, 24 studies met the inclusion criteria for meta-analysis (16–39). A manual reference search identified seven additional potentially relevant studies, all of which had already been captured in the original electronic search. The complete study selection process is illustrated in Figure 1.

### Study characteristics

Table 1 summarizes the baseline characteristics of the included studies and participants. The 24 studies involved 3,509 participants, with 23 conducted in China and one in the United States. The mean age of enrolled participants ranged from 48.6 to 68.0 years, with male representation ranging from 46.1 to 70.8%. Nine studies exclusively included confirmed CHD participants, while the remaining 15 included those with suspected CHD. Coronary angiography (CAG) was used as the reference standard in 18 studies, while myocardial perfusion imaging (MPI) was employed in six. Methodological quality assessment, detailed in Table 2, indicated moderate to high quality across all studies, with overall high methodological rigor.

**Table 1:** The baseline characteristics of identified studies and involved patients.

Study	Region	Sample size	Age (years)	Male (%)	Participants status	Gold standard	TP	FP	FN	TN
Zhang 2014 (16)	China	90	62.8	55.6	CHD	CAG	55	12	10	13
Shan 2015 (17)	China	88	61.0	51.1	Suspected CHD	CAG	36	6	21	29
Wang 2015 (18)	China	55	48.6	67.3	CHD	CAG	16	5	11	12
Wang 2016 (19)	China	120	63.0	60.0	Suspected CHD	CAG	44	23	24	29
Xie 2017 (20)	China	96	59.8	70.8	Suspected CHD	CAG	56	3	28	9
Dong 2017 (21)	China	55	54.2	69.1	Suspected CHD	MPI	23	6	10	16
Liu 2017 (22)	China	300	54.9	64.7	Suspected CHD	MPI	132	26	53	89
Tang 2018 (23)	China	112	62.8	51.8	Suspected CHD	CAG	61	6	35	9
Pelter 2018 (24)	USA	361	63.0	62.0	Suspected CHD	CAG	113	112	34	102
He 2019 (25)	China	62	58.7	51.6	CHD	CAG	32	1	13	16
Dong 2019 (26)	China	152	58.8	55.3	Suspected CHD	CAG	56	18	32	46
Li 2019 (27)	China	63	67.4	57.1	CHD	CAG	35	5	6	17
Nan 2019 (28)	China	160	61.8	60.0	CHD	CAG	132	2	6	18
Ye 2019 (29)	China	150	61.9	60.7	CHD	CAG	100	7	10	33
Wen 2019 (30)	China	120	59.2	55.8	Suspected CHD	MPI	58	10	22	30
Wu 2020 (31)	China	100	68.0	49.0	Suspected CHD	MPI	48	21	22	9
Fu 2020 (32)	China	562	64.9	60.0	Suspected CHD	CAG	213	101	115	133
Cheng 2020 (33)	China	78	56.0	60.3	Suspected CHD	CAG	23	15	7	33
Ren 2020 (34)	China	163	56.3	55.8	Suspected CHD	CAG	64	30	29	40
He 2021 (35)	China	102	54.2	68.6	Suspected CHD	MPI	43	11	18	30
Chen 2021 (36)	China	158	51.3	69.6	CHD	MPI	48	22	31	57
Li 2021 (37)	China	80	65.3	66.3	CHD	CAG	43	7	5	25
Chen 2022 (38)	China	206	65.6	54.9	Suspected CHD	CAG	150	12	24	20
Qiu 2024 (39)	China	76	50.0–66.0	46.1	CHD	CAG	46	2	14	14

*CAG, coronary arteriography; CHD, coronary heart disease; FN, false negative; FP, false positive; MPI, myocardial perfusion imaging; TN, true negative; TP, true negative.

**Table 2:** The methodological quality of included studies.

Study	Risk of bias				Applicability concerns		
	Patient selection	Index test	Reference standard	Flow and timing	Patient selection	Index test	Reference standard
Zhang 2014 (16)	Low	Low	Unclear	Low	Low	Low	Unclear
Shan 2015 (17)	Unclear	Low	Low	Low	Unclear	Low	Low
Wang 2015 (18)	Low	Low	Unclear	Low	Low	Low	Unclear
Wang 2016 (19)	Low	Low	Unclear	Low	Low	Low	Unclear
Xie 2017 (20)	Low	Low	Low	Low	Low	Low	Low
Dong 2017 (21)	Low	Low	Low	Low	Low	Low	Low
Liu 2017 (22)	Low	Low	Low	Low	Low	Low	Low
Tang 2018 (23)	Low	Low	Low	Low	Low	Low	Low
Pelter 2018 (24)	Low	Low	Low	Low	Low	Low	Low
He 2019 (25)	Unclear	Low	Low	Low	Low	Low	Low
Dong 2019 (26)	Unclear	Low	Low	Unclear	Unclear	Low	Low
Li 2019 (27)	Low	High	Unclear	Low	Low	High	Unclear
Nan 2019 (28)	Low	Low	Low	Low	Low	Low	Low
Ye 2019 (29)	Low	Low	Low	Low	Low	Low	Low
Wen 2019 (30)	Low	Unclear	Low	Low	Low	Unclear	Low
Wu 2020 (31)	Unclear	Low	Low	Low	Unclear	Low	Low
Fu 2020 (32)	Low	Low	Unclear	Low	Low	Low	Low
Cheng 2020 (33)	Low	Low	Low	Unclear	Low	Low	Low
Ren 2020 (34)	Low	Low	Low	Low	Low	Low	Low
He 2021 (35)	Unclear	Low	Low	Low	Unclear	Low	Low
Chen 2021 (36)	High	Low	Low	Low	High	Low	Low
Li 2021 (37)	Unclear	Low	Low	Low	Unclear	Low	Low
Chen 2022 (38)	Low	Low	Low	Low	Low	Low	Low
Qiu 2024 (39)	Unclear	Low	Low	Low	Unclear	Low	Low

### Sensitivity and specificity

**Figure 2:**
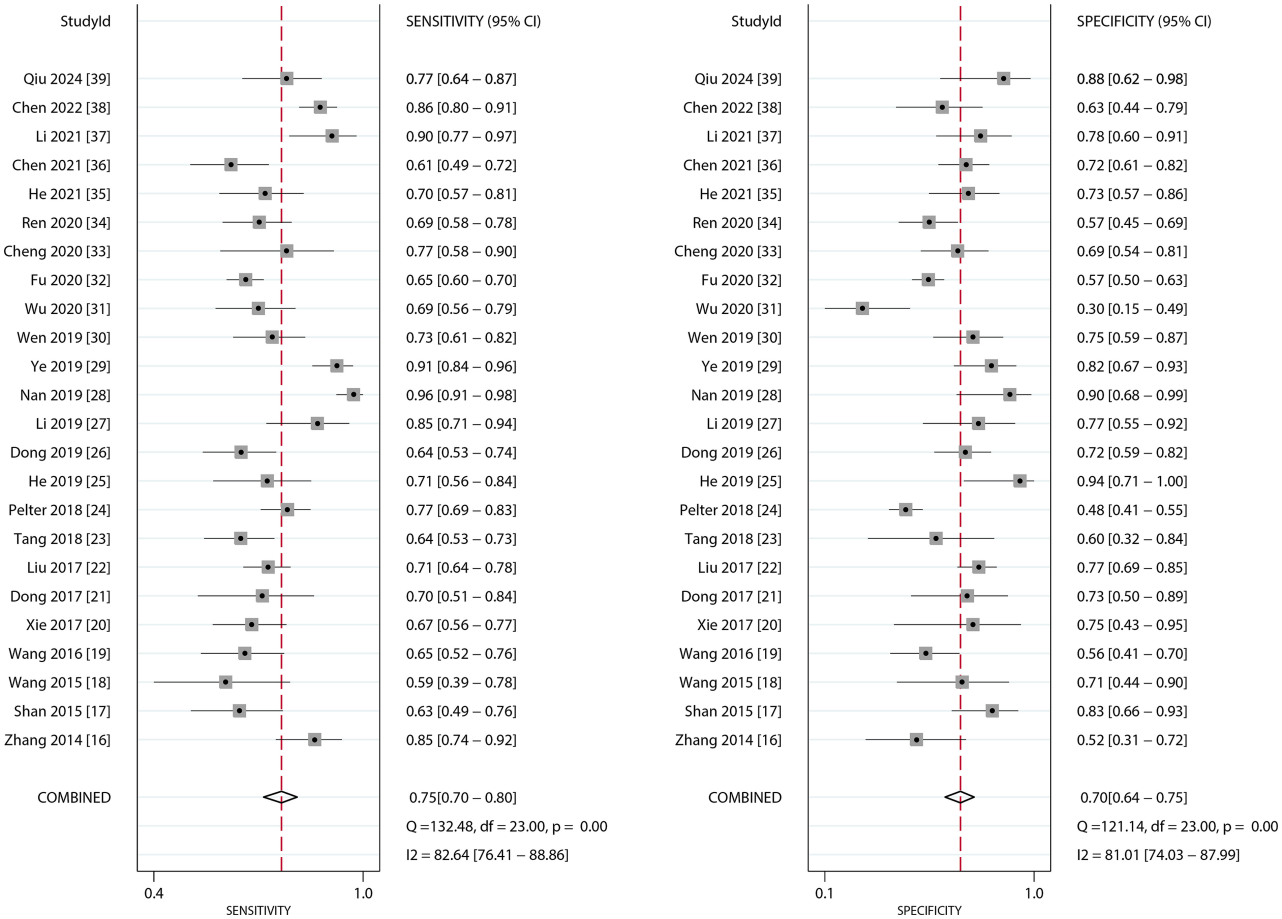
The summary sensitivity and specificity of dynamic ECG for detecting myocardial ischemia among CHD patients.

Figure 2 presents the pooled diagnostic performance of dynamic ECG in detecting myocardial ischemia among participants with CHD, demonstrating a sensitivity of 0.75 (95% CI: 0.70–0.80) and specificity of 0.70 (95% CI: 0.64–0.75). Significant heterogeneity was observed for both metrics (*I^2^* ≥ 50%, *p* < 0.10). Subgroup analyses showed higher sensitivity and specificity in studies involving confirmed CHD participants compared to those with suspected CHD, with statistically significant between-subgroup differences. Diagnostic performance was also higher when CAG was used as the reference standard, although no significant subgroup difference in specificity was observed (Table 3).

**Table 3:** Subgroup analyses for the diagnostic performance of dynamic electrocardiography for detecting myocardial ischemic attack in coronary heart disease.

Diagnostic metrics	Factors	Subgroups	ES and 95%CI	*I^2^* (%)	Difference between subgroups
Sensitivity	Disease status	Suspected CHD	0.70 (0.66–0.74)	62.40	0.84 (0.75–0.95)
		Confirmed CHD	0.83 (0.73–0.89)	87.52	
	Gold standard	CAG	0.77 (0.71–0.83)	87.08	1.12 (1.01–1.23)
		MPI	0.69 (0.65–0.73)	0.00	
Specificity	Disease status	Suspected CHD	0.65 (0.58–0.71)	78.75	0.83 (0.72–0.96)
		Confirmed CHD	0.78 (0.70–0.85)	60.24	
	Gold standard	CAG	0.70 (0.63–0.76)	81.53	1.03 (0.84–1.26)
		MPI	0.68 (0.55–0.79)	81.36	
PLR	Disease status	Suspected CHD	1.99 (1.63–2.42)	65.66	0.53 (0.35–0.80)
		Confirmed CHD	3.77 (2.60–5.47)	57.41	
	Gold standard	CAG	2.58 (1.98–3.37)	79.18	1.18 (0.73–1.89)
		MPI	2.19 (1.48–3.25)	78.47	
NLR	Disease status	Suspected CHD	0.46 (0.39–0.54)	60.84	2.09 (1.25–3.49)
		Confirmed CHD	0.22 (0.14–0.37)	88.02	
	Gold standard	CAG	0.33 (0.24–0.44)	87.33	0.73 (0.50–1.07)
		MPI	0.45 (0.36–0.57)	57.09	
DOR	Disease status	Suspected CHD	4.29 (3.07–6.00)	66.70	0.24 (0.09–0.62)
		Confirmed CHD	17.69 (7.34–42.63)	78.70	
	Gold standard	CAG	7.73 (4.80–12.44)	80.80	1.63 (0.74–3.57)
		MPI	4.75 (2.54–8.89)	72.40	
AUC	Disease status	Suspected CHD	0.73 (0.69–0.77)	–	0.85 (0.79–0.91)
		Confirmed CHD	0.86 (0.82–0.89)	–	
	Gold standard	CAG	0.80 (0.76–0.83)	–	1.14 (1.06–1.23)
		MPI	0.70 (0.66–0.74)	–	

### PLR and NLR

**Figure 3:**
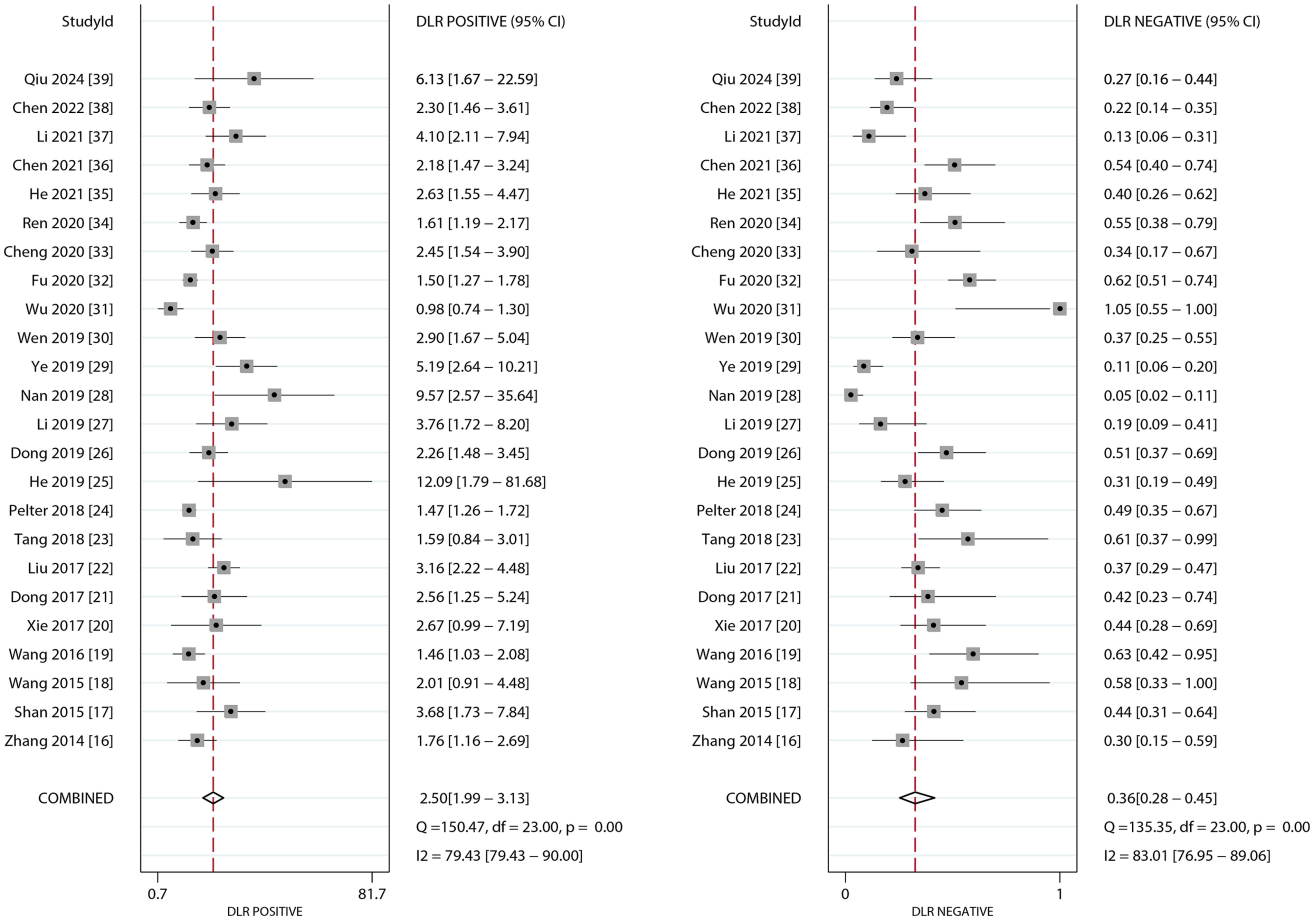
The summary PLR and DLR of dynamic ECG for detecting myocardial ischemia among CHD patients.

Figure 3 presents the pooled results for PLR and NLR of dynamic ECG in diagnosing myocardial ischemia among participants with CHD. The pooled PLR was 2.50 (95% CI: 1.99–3.13), and the NLR was 0.36 (95% CI: 0.28–0.45). Significant heterogeneity was found for both metrics (*I^2^* ≥ 50%, *p* < 0.10). Subgroup analyses revealed higher PLR values in studies involving confirmed participants with CHD and those using CAG as the reference standard. Statistically significant subgroup differences were observed for participant status (*p* < 0.05), but not for reference standard methodology. Conversely, NLR values were lower in confirmed CHD participants and CAG-based studies, with significant differences by participant status (*p* < 0.01) but not across reference standards (Table 3).

### DOR

**Figure 4:**
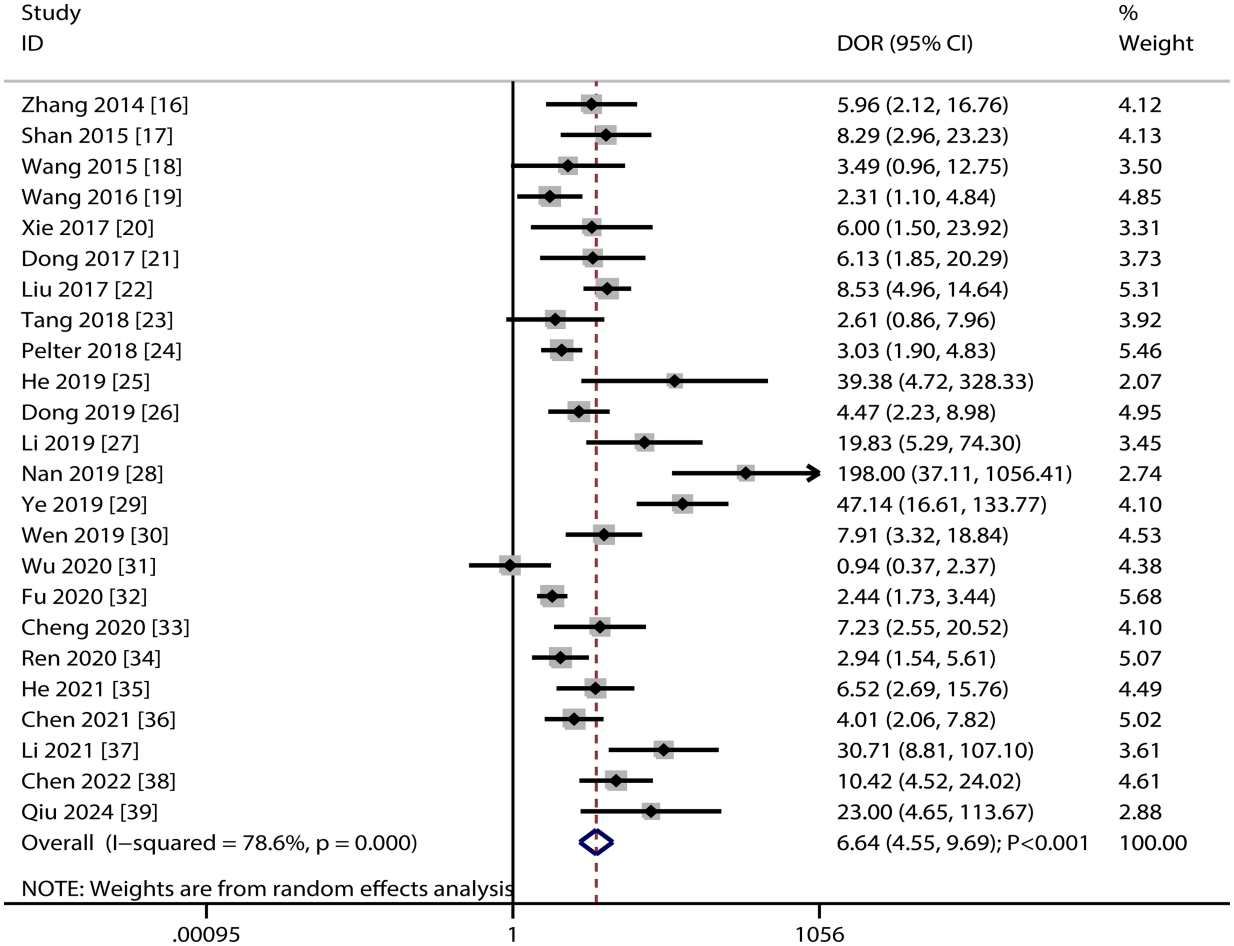
The summary DOR of dynamic ECG for detecting myocardial ischemia among CHD patients.

Figure 4 presents the pooled DOR results for dynamic ECG in detecting myocardial ischemia among participants with CHD, showing a DOR of 6.64 (95% CI: 4.55–9.69). Significant heterogeneity was observed in the DOR estimates (I^2^ ≥ 50%, *p* < 0.10). Subgroup analyses demonstrated elevated DOR values in studies involving confirmed CHD participants and those using CAG as the reference standard. Statistically significant between-subgroup differences were found for participant status (*p* < 0.05), while no significant variation was observed across reference standard subgroups (Table 3).

### AUC

**Figure 5:**
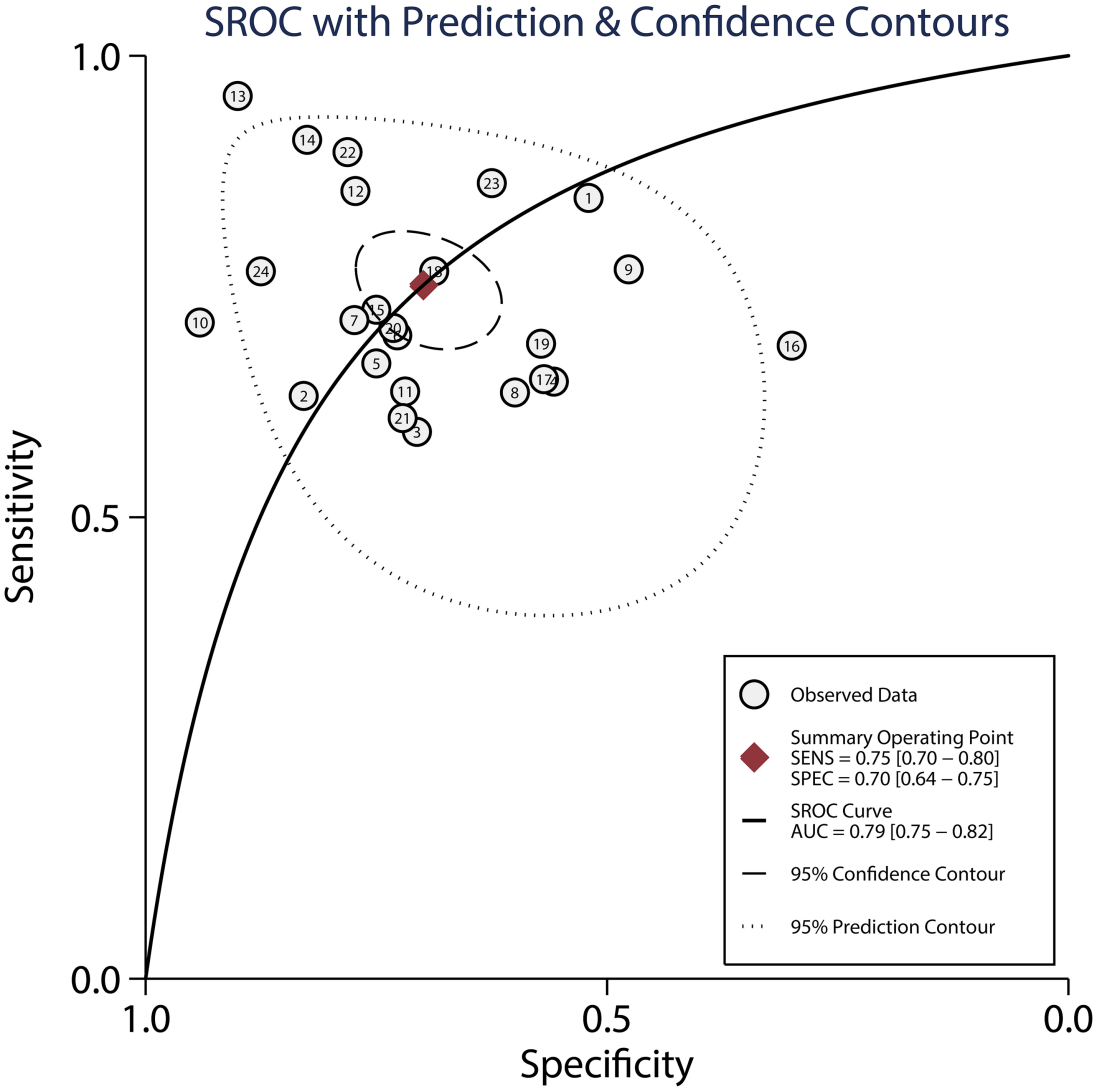
The summary area under the receiver operating characteristic curve of dynamic ECG for detecting myocardial ischemia among CHD patients.

Figure 5 presents the pooled AUC results for dynamic ECG in detecting myocardial ischemia among participants with CHD, demonstrating an AUC of 0.79 (95% CI: 0.75–0.82). Subgroup analyses revealed significantly higher AUC values in studies involving confirmed CHD participants and those using CAG as the reference standard, with statistically significant between-subgroup differences (*p* < 0.05), as shown in Table 3.

### Publication bias

**Figure 6:**
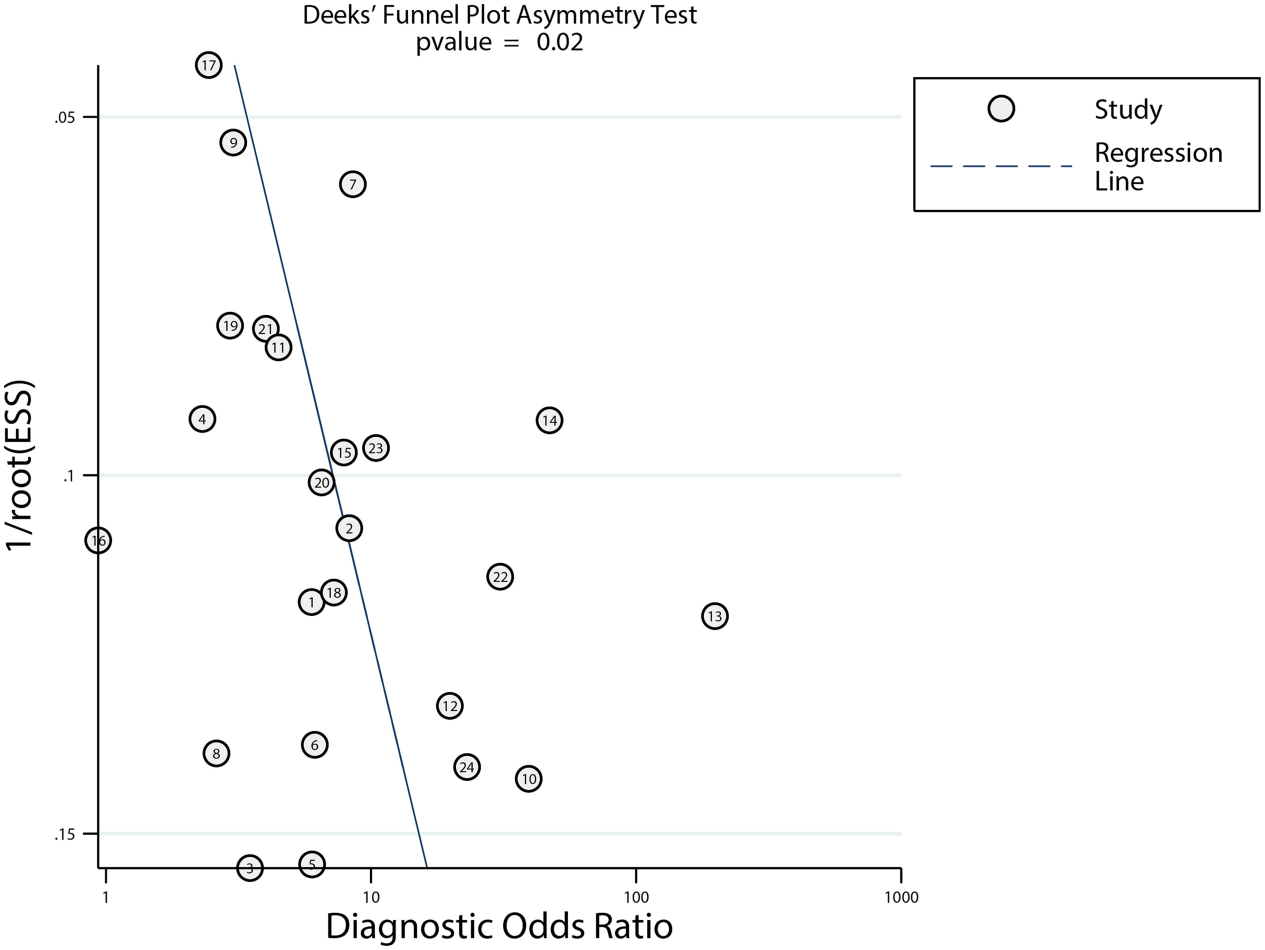
Funnel plots with Deeks’ asymmetry tests of dynamic ECG for detecting myocardial ischemia among CHD patients.

Visual inspection of the funnel plot could not rule out potential publication bias (Figure 6). Deeks’ asymmetry test indicated significant publication bias in the diagnostic performance of dynamic ECG for myocardial ischemia detection (*p* = 0.02). We conducted a trim-fill analysis to adjust for this bias, After adjusting potential publication bias using the trim and fill method, the pooled diagnostic metrics remained consistent. These adjusted values confirm that while publication bias may modestly overestimate accuracy, the overall pattern of moderate diagnostic utility remains unchanged.

## Discussion

This systematic review and meta-analysis provides a comprehensive evaluation of dynamic ECG for detecting myocardial ischemia in participants with CHD. The pooled diagnostic estimates—sensitivity, 0.75; specificity, 0.70; PLR, 2.50; NLR, 0.36; DOR, 6.64 and AUC, 0.79—indicate moderate diagnostic utility. While clinically relevant, these values fall short of ideal diagnostic thresholds, highlighting the need for complementary diagnostic tools (40). A PLR of 2.50 suggests that a positive dynamic ECG increases the post-test probability of myocardial ischemia by approximately 30% in moderate-prevalence populations, while an NLR of 0.36 decreases the probability by 40–50%. These findings support the use of dynamic ECG as a triage tool rather than a definitive diagnostic method, consistent with its established role in ambulatory monitoring of transient ischemic episodes (40).

The high heterogeneity (*I^2^* > 80% for sensitivity and specificity) is multifactorial, with unreported methodological details emerging as a critical challenge. First, population heterogeneity—including differences in age, sex distribution (46.1–70.8% male), and comorbidities such as diabetes—may have contributed to inconsistent ischemic patterns, as diabetes alters ST-segment morphology through autonomic dysfunction (41). Second, variation in ischemic threshold definitions (e.g., ST-segment depression criteria: ≥0.1 mV vs. ≥1 mm; duration: ≥1 min vs. transient episodes) introduces diagnostic inconsistency, as minor differences in cutoff values can significantly affect sensitivity and specificity trade-offs (42). Third, differences in lead configurations and artifact discrimination algorithms impact signal fidelity, particularly during patient movement—a known limitation of ambulatory monitoring. Finally, use of different reference standards introduces spectrum bias: CAG directly visualizes coronary stenosis, while MPI evaluates functional ischemia, capturing distinct pathophysiological processes.

Subgroup analyses demonstrated enhanced diagnostic performance in confirmed participants with CHD (vs. suspected cases) and CAG-based studies. The superior performance in confirmed CHD cohorts likely reflects greater atherosclerotic burden, allowing dynamic ECG to more reliably detect ischemia-induced repolarization abnormalities (43). In contrast, participants with suspected CHD may exhibit non-ischemic ST-T changes resulting from conditions such as microvascular dysfunction or electrolyte imbalance, reducing specificity. The improved performance in CAG-based studies underscores the value of anatomical correlation, as transient ECG changes may not align with perfusion defects seen in MPI—particularly in cases of balanced multivessel disease (44, 45). However, the absence of significant specificity differences across reference standards indicates ongoing challenges in distinguishing true ischemic events from physiological confounders.

Significant publication bias (Deeks’ test *p* = 0.02) suggests that smaller studies with less favorable diagnostic performance may have been underreported, a common issue in diagnostic meta-analyses (46). Several factors likely contribute: (1) researchers and journals may be more inclined to publish studies with “positive” findings, while studies with non-significant or lower accuracy are less likely to be submitted or accepted. This is particularly relevant for dynamic ECG research, where institutional or commercial interests in validating diagnostic tools may influence publication trends; (2) our trim-fill analysis imputed five hypothetical missing studies, which slightly reduced pooled metrics but preserved the conclusion of moderate diagnostic utility. This suggests the overestimation due to bias is modest rather than transformative; and (3) while we cannot access unpublished data, we can infer their potential characteristics: smaller sample sizes, higher risk of bias, or populations with lower disease prevalence—factors known to reduce diagnostic metric precision. Inclusion of such studies would likely widen confidence intervals but not negate the core finding that dynamic ECG has clinical utility for ischemia detection.

A notable limitation of this meta-analysis is the overrepresentation of Chinese studies (23/24), which may restrict the extrapolability of conclusions to other populations. Several factors may explain the paucity of international studies meeting our criteria: (1) Clinical practice variations: Dynamic ECG utilization patterns differ globally—while it is widely adopted as a first-line ambulatory monitoring tool in China for CHD patients, international guidelines often prioritize stress testing or coronary CT angiography for ischemia detection, potentially reducing the number of dedicated dynamic ECG diagnostic studies; and (2) Data reporting standards: International guideline focus on dynamic ECG for arrhythmia detection rather than myocardial ischemia, or lack complete diagnostic data required for meta-analysis. Thus, while our findings provide valuable evidence for Chinese clinical practice, extrapolation to other regions should be cautious. Future studies should prioritize multi-center, international collaborations to include diverse ethnicities, healthcare systems, and clinical practice patterns, thereby enhancing the generalizability of dynamic ECG’s diagnostic performance data. Moreover, the lack of standardized reporting of ST-segment depression thresholds and duration criteria in mostly included studies. Threshold variations are well-known to drive sensitivity-specificity trade-offs in diagnostic testing. Without this data, we cannot quantify their contribution to heterogeneity, highlighting a major gap in dynamic ECG research: the absence of consensus on reporting diagnostic criteria.

## Conclusion

Despite limitations—including significant geographic bias (23 of 24 studies from China) and methodological heterogeneity—these findings reinforce the utility of dynamic ECG in non-invasive ischemia monitoring. The consistency between the single U. S. study and pooled Chinese results provides preliminary support for generalizability, but future international studies are needed to confirm these findings across diverse populations. Its strength lies in capturing transient episodes during routine activity, offering a preferable alternative to stress testing in older adults and patients with frailty. Clinicians should interpret results within the broader clinical context. Future research should prioritize standardization of ischemic criteria, adoption of advanced signal-processing technologies, and validation across diverse populations. Integrating dynamic ECG with high-sensitivity troponin assays or coronary CT angiography may enhance diagnostic accuracy, particularly in emergency department evaluations for chest pain.

## Data Availability

The original contributions presented in the study are included in the article/Supplementary material, further inquiries can be directed to the corresponding author.
