# Supplementary material for: Diagnostic performance of dynamic electrocardiography in the diagnosis of myocardial ischemic attack in coronary heart disease: a systematic review and meta-analysis
Source: Front Med (Lausanne). 2025 Sep 17;12:1646417. doi: 10.3389/fmed.2025.1646417 (PMC12484157; doi:10.3389/fmed.2025.1646417)
Supplement: Supplementary file 1 [file Table_1.docx]

**PubMed:**

#1. ("Electrocardiography, Ambulatory"[Mesh] OR "Holter Monitoring"[Mesh]) AND ("Myocardial Ischemia"[Mesh] OR "Coronary Disease"[Mesh])

#2. (“dynamic ECG” OR “ambulatory electrocardiogram” OR “Holter ECG”) AND (“myocardial ischemia” OR “coronary heart disease” OR “CHD”) AND (“diagnosis” OR “diagnostic accuracy” OR “sensitivity” OR “specificity”)

#3. #1 or #2.

**EmBase:**

#1. (“ambulatory electrocardiography”/exp OR “Holter monitoring”/exp) AND (“myocardial ischaemia”/exp OR “coronary artery disease”/exp)

#2. (“dynamic ECG” OR “ambulatory electrocardiogram” OR “Holter ECG”) AND (“myocardial ischemia” OR “myocardial ischaemia” OR “coronary heart disease” OR “coronary artery disease” OR “CHD”) AND (“diagnosis” OR “diagnostic accuracy” OR “sensitivity” OR “specificity”)

#3. #1 or #2.

**Web of Science:**

TS = (“dynamic ECG” OR “ambulatory electrocardiogram” OR “Holter ECG”) AND TS = (“myocardial ischemia” OR “coronary heart disease” OR “CHD”) AND TS = (“diagnosis” OR “diagnostic accuracy” OR “sensitivity” OR “specificity”)

**Cochrane Library:**

#1 “dynamic ECG” OR “ambulatory electrocardiogram” OR “Holter ECG”

#2 “myocardial ischemia” OR “coronary heart disease” OR “CHD”

#3 “diagnosis” OR “diagnostic accuracy” OR “sensitivity” OR “specificity”

#4 #1 AND #2 AND #3

**China National Knowledge Infrastructure:**

SU = （动态心电图 OR 动态心电监测 OR 动态心电记录） AND SU = （心肌缺血 OR 冠心病） AND SU = （诊断 OR 诊断准确性 OR 敏感度 OR 特异度）

**Wanfang:**

（动态心电图 + 动态心电监测 + 动态心电记录） AND 主题:（心肌缺血 + 冠心病） AND 主题:（诊断 + 诊断准确性 + 敏感度 + 特异度）
